# When to germinate: the talk between abscisic acid and circadian clock

**DOI:** 10.1093/plphys/kiad014

**Published:** 2023-01-17

**Authors:** Kaikai Zhu, Yajin Ye

**Affiliations:** College of Forestry, Nanjing Forestry University, Nanjing, Jiangsu 210037, China; College of Forestry, Nanjing Forestry University, Nanjing, Jiangsu 210037, China

As an important plant behavior regulator, the circadian clock enables plants to participate in and synchronize multiple physiological and developmental responses, including stress responses (Sanchez and Kay, 2016). Abscisic acid (ABA) is also a key regulator in plant growth, development, and stress responses (Weiner et al., 2010). Responses regulated by the circadian oscillator also modulate clock performance. These feedback loops and their multilayer communications create a complex network to regulate the acclimation of plants to the environment (Sanchez and Kay, 2016). A close relationship between the circadian clock and the ABA pathway has been reported (Yang et al., 2021); however, the feedback regulation loops between them are poorly understood (Seo and Mas, 2015; Yoshida et al., 2019).

In this issue of *Plant Physiology*, Wang et al. (2022) discovered a crosstalk between ABA signaling and the circadian clock in rice (*Oryza sativa*). This crosstalk module includes ABA receptor family member regulatory components of ABA receptor 10 (RCAR10), ABA-signaling pathway transcription factor ABA insensitive 5 (ABI5), and clock component pseudo-response regulator 95 (PRR95). Their results demonstrated that RCAR10-ABI5-PRR95 functions in a feedback loop to modulate ABA signaling and thus fine-tune seed germination and seeding growth (Wang et al., 2022).

To identify genetic components involved in ABA responses, the authors created a rice CRISPR/Cas9 mutant library targeting 134 transcription factors of interest and screened this library for mutants with germination defects. Consequently, a mutant of *OsPRR95* was isolated that displayed delayed germination and seedling growth compared with its wild type. Moreover, the *osprr95* mutant showed hypersensitivity to exogenous ABA treatment. Similarly, compared with the wild-type control, seed germination, and seedling growth of the mutants were more severely inhibited by salt and osmotic stress, indicating OsPRR95 regulates seed germination and seedling growth in response to salt stress and osmotic stress (Wang et al., 2022).

What is the molecular mechanism by which OsPRR95 regulates ABA pathways? The authors first analyzed the expression level of key genes involved in ABA biosynthetic, catabolic, and signaling pathways. Compared with the wild type, the expression levels of ABA-responsive genes and ABA-signaling-related genes were significantly increased in *osprr95*, indicating that the enhanced ABA sensitivity of *osprr95* was mainly due to increased ABA signaling.

Given the fact that the gene encoding ABA receptor OsRCAR10 displayed an opposite rhythmic expression compared with *OsPRR95*, the authors tested the possibility of the direct regulation of OsPRR95 on *OsRCAR10*. Gel shift, ChIP-qPCR, and transient expression assays all demonstrated that OsPRR95 represses *OsRCAR10* expression through direct binding to its promoter. In addition, with regard to seed germination and seedling growth, the *osprr95 osrcar10* double mutant showed similar sensitivity as the *osrcar10* mutant to exogenous ABA treatment, suggesting that OsPRR95 acts upstream of OsRCAR10. Intriguingly, compared with wild type, ABA-induced *OsPRR95* expression in the *OsRCAR10* overexpression line was significantly higher, suggesting OsRCAR10 is involved in the induction of *OsPRR95* by ABA.

Since the responses regulated by the circadian clock are usually able to modulate clock performance through feedback (Seo and Mas, 2014; Srivastava et al., 2019), the authors explored the possible effects of ABA signaling on *OsPRR95* expression. Taking cues from the experimental results that ABA could induce *OsPRR95* expression, the authors discovered that the key ABA-signaling transcription factor ABI5 activates *OsPRR95* expression through direct binding to the *OsPRR95* promoter using gel shift, ChIP-qPCR, and transient expression assays and thus establishing a direct regulatory module from ABA signaling to a core circadian oscillator.

**Figure 1:**
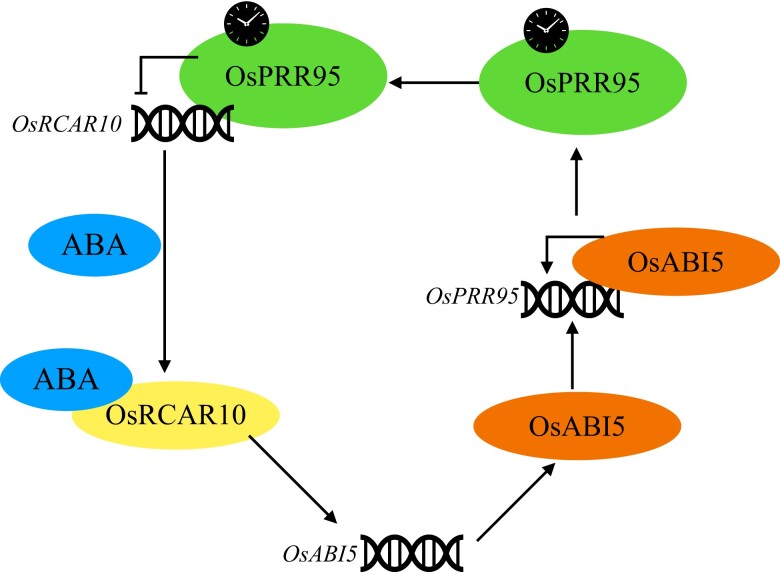
The RCAR10-ABI5-PRR95 feedback loop in rice. OsPRR95 binds to *OsRCAR10* directly and represses *OsRCAR10* expression. OsRCAR10 induces the expression of *OsABI5*, and OsABI5 further binds directly to the promoter region of *OsPRR95* to activate its expression, forming the RCAR10-ABI5-PRR95 feedback regulatory loop. Adapted from Wang et al. (2022).

In summary, the work presented here advances our understanding of the feedback regulation loop between ABA signaling and the circadian clock during seed germination, which is achieved through the RCAR10-ABI5-PRR95 regulatory module (Figure 1). The circadian clock controls many aspects of plant growth and environmental responses, and previous studies have shown a link between the clock and stress signaling and ABA responses (Seo and Mas, 2014; Li et al., 2022; Oravec and Greenham, 2022). The work by Wang et al. shows a direct point of integration between these two key pathways. As the demand for stress-resistant crops becomes increasingly urgent, further studies on this crosstalk could lead to new insights to develop stress-resistant crops. Here, Wang and collaborators have set the starting point.
